# Interval-valued Pythagorean fuzzy multi-criteria decision-making method based on the set pair analysis theory and Choquet integral

**DOI:** 10.1007/s40747-022-00778-7

**Published:** 2022-06-17

**Authors:** Feng Li, Jialiang Xie, Mingwei Lin

**Affiliations:** 1 School of Science, Jimei University, Xiamen, 361021 Fujian China; 2 College of Computer and Cyber Security, Fujian Normal University, Fuzhou, 350117 Fujian China

**Keywords:** Interval-valued Pythagorean fuzzy set, Set pair analysis, Connection number, Multi-criteria decision-making, Choquet integral

## Abstract

This paper proposes a novel fuzzy multi-criteria decision-making method based on an improved score function of connection numbers and Choquet integral under interval-valued Pythagorean fuzzy environment. To do so, we first introduce a method to convert interval-valued Pythagorean fuzzy numbers into connection numbers based on the set pair analysis theory. Then an improved score function of connection numbers is proposed to make the ranking order of connection numbers more in line with reality in multi-criteria decision-making process. In addition, some properties of the proposed score function of connection numbers and some examples have been given to illustrate the advantages of conversion method proposed in the paper. Then, considering interactions among different criteria, we propose a fuzzy multi-criteria decision-making approach based on set pair analysis and Choquet integral under interval-valued Pythagorean fuzzy environment. Finally, a case of online learning satisfaction survey and a brief comparative analysis with other existing approaches are studied to show that the proposed method is simple,convenient and easy to implement. Comparing with previous studies, the method in this paper, from a new perspective, effectively deals with multi-criteria decision-making problems that the alternatives cannot be reasonably ranked in the decision-making process under interval-valued Pythagorean fuzzy environment.

## Introduction

An important issue in multi-criteria decision-making (MCDM) is to obtain a reasonable ranking order of all alternatives. Due to the complexity of reality, fuzzy and uncertain information is naturally involved in MCDM process. For this reason, the theory of fuzzy set [1], intuitionistic fuzzy set (IFS) [2], interval-valued intuitionistic fuzzy set (IVIFS) [3] and their applications have been put forward one after another with the development of research [4–10]. However, Yager [11] proposed such an example in real life: a decision maker may express his satisfaction with an alternative on a criterion is 0.6, but his dissatisfaction is 0.5. Because $$ 0.6 + 0.5 > 1$$, the above special case cannot be modeled by the theory of IFS or IVIFS , which requires the sum of membership degree and the non-membership degree less than or equal to one [12]. Therefore, a concept of Pythagorean fuzzy set (PFS) is introduced by Yager, of which the square sum of membership degree and non-membership degree is less than or equal to one [11]. As extensions of PFS, Smarandache [13] introduced the refined Pythagorean fuzzy sets, Ünver [14] defined Spherical Fuzzy Sets and Zhang [15] proposed a concept of interval-valued Pythagorean fuzzy set (IVPFS). As powerful tools to deal with vagueness and uncertainty involved in MCDM problems, theories and applications of these sets have recently been extensively studied in the literature. For instance, Ejegwa [16] solved career placement problems under the Pythagorean fuzzy environment. Saeed et al. [17] showed the properties, set-theoretic operations and axiomatic results for the refined Pythagorean Fuzzy Sets. For more details, please refer to [18–26].

It is noteworthy that the ranking technique of fuzzy numbers is crucial in the fuzzy MCDM process [15]. That is, if the ranking technique is not appropriate then no matter what fuzzy MCDM method you use, the results are unreliable or even completely inconsistent with reality [27]. On the other hand, the interval-valued Pythagorean fuzzy numbers (IVPFNs) involves more uncertainties than other fuzzy numbers (e.g., intuitionistic fuzzy numbers (IFNs), interval-valued intuitionistic fuzzy numbers (IVIFNs), Pythagorean fuzzy numbers (PFNs),etc) which are usually able to adapt to higher degrees of uncertainty [28]. In order to make the solutions to MCDM problems more reliable, it is necessary to develop a ranking method which not only ranks IVPFNs intuitively but also loses useful information as little as possible [27]. Ever since IVPFNs’ appearance, many studies have focused on the ranking problems under interval-valued Pythagorean fuzzy environment. For instance, Zhang [15] proposed a ranking method based on the closeness index of PFNs and IVPFNs and presented a Pythagorean fuzzy hierarchical qualitative flexible multiple criteria approach (QUALIFLEX) to solve the fuzzy MCDM problems. Although the method is relatively simple, it relies too much on the definition of distance for PFNs and IVPFNs. That is, different distances will get different ranking results which will bring some inconveniences in fuzzy MCDM process. Moreover, it is noteworthy that the score function and the accuracy function are important tools for ranking PFNs and IVPFNs [15]. Zhang [15] introduced the score function and accuracy function of IVPFNs, which generalized the definition for PFNs in [29]. However, these definitions will lead to a certain loss of information, because they failed to consider the influence of hesitation of IVPFNs in fuzzy MCDM process under the interval-valued Pythagorean fuzzy environment. Therefore, a novel accuracy function of IVPFNs [30], an improved accuracy function [12] and an improved score function of IVPFNs [28] were proposed by Garg considering the effect of hesitation interval index of IVPFNs. These above ranking methods have been widely used in the field of interval-valued Pythagorean fuzzy MCDM.

However, by browsing the literature, we find that there is one type of IVPFNs, the elements in which cannot be reasonably ranked in MCDM process using existing methods. This type of IVPFNs satisfies the following two properties: first, for each IVPFN, the lower limit of its membership degree is equal to the lower limit of its non-membership degree, and the upper limit of its membership degree is equal to the upper limit of its non-membership degree; second, the square sum of lower limit and upper limit of the membership degree of the one set is equal to the square sum of lower limit and upper limit of the membership degree of the other one. According to the existing methods, it can be concluded that these sets are equivalent even if they are completely different ones (see *Example* 4 for details). In addition, we find the value of improved accuracy function for some IVPFNs may exceed one even if it is not the largest IVPFN (see *Example* 5 for details). Obviously, all these results are not in line with reality. In view of the above analysis, it is necessary to propose a new ranking approach for IVPFNs from a new perspective to obtain a reasonable order between them.

The set pair analysis (SPA) theory is a new framework combining the certainty and uncertainty into a unified way [31]. The connection number (CN) is a principal mathematical tool of SPA [32]. It uses the degree of ’identity’, ’discrepancy’, and ’contrary’ to indicate the certainty, hesitancy, and uncertainty of a system, respectively [33]. Since the SPA theory was proposed, researchers have done a lot of in-depth studies on its theory and applications under IFS and IVIFS environment [34–37]. For example, based on the SPA theory, Garg and Kumar proposed some similarity measures of IFSs [38] and some series of distance measures for IFSs [39]. In [40], they introduced a TOPSIS IVIFS MADM method in decision-making process using the SPA theory. And to rank different IVIFNs, Garg and Kumar proposed a new possibility measure of IVIFS based on the CNs of SPA [41]. Kumar and Chen [42] proposed a multi-attribute decision making method based on SPA under the interval-valued intuitionistic fuzzy environment and introduced a score function of connection numbers, and so on. Since IVPFS is the generalization of IVIFS, it can be inferred that the SPA theory can also be a useful tool to deal with uncertainty in MCDM process under interval-valued Pythagorean fuzzy environment. Unfortunately, we have not yet found any application of the SPA theory under the interval-valued Pythagorean fuzzy environment, let alone the research on ranking techniques and multi-attribute decision making methods under the interval-valued Pythagorean fuzzy environment. Moreover, the proposed score function of CNs [42] has some shortcomings which are unable to get the reasonable sorting of alternatives in some MCDM processes under the interval-valued Pythagorean fuzzy environment (for details, see *Example*2).

Motivated by above analysis, this paper first develops a novel ranking method for IVPFNs based on the SPA theory. That is, in order to get a reasonable order of IVPFNs in MCDM process, a technique to convert IVPFNs into CNs based on SPA is introduced at first by taking the hesitation interval index and Pythagorean property of IVPFNs into consideration properly. Then we propose an improved score function of CNs which can make the ranking order of CNs more in line with reality. The properties of the proposed score function of CNs and some examples are also given to illustrate the advantage of our proposed ranking method. Next, considering interactions among different criteria in the decision-making process, we propose a fuzzy MCDM approach under interval-valued Pythagorean fuzzy environment based on SPA and Choquet integral which is used to aggregate the evaluation information of criteria for each alternative.

Therefore, the innovations of this paper are summarized as follows :

(i) A novel technique of converting IVPFNs into CNs based on SPA is proposed to rank IVPFNs in MCDM process from a new perspective for the first time in the literature. In addition, the idea of transformation fully takes into account the influence of hesitation interval index and Pythagorean property on information uncertainty under the interval-valued Pythagorean fuzzy environment.
(ii) An improved score function of CNs is presented which can make the ranking order of CNs more in line with reality, and overcome the shortcomings of existing formulas;
(iii) The aggregation of alternative evaluation information using SPA and Choquet integral considers uncertainty and interactions among criteria in MCDM under interval-valued Pythagorean fuzzy environment simultaneously.

This paper is organized as follows. The basic concepts of PFS, IVPFS, the SPA theory, the score function and accuracy function of CNs as well as fuzzy measure and Choquet integral are reviewed in the next section. The technique to convert IVPFNs into CNs and an improved score function of CNs are proposed and some examples are given to verify the advantage of the proposed converting method from IVPFNS into CNs in the following section. A novel MCDM approach based on SPA and Choquet integral under interval-valued Pythagorean fuzzy environment is introduced in the next section. An example of online learning satisfaction survey and a brief discussion and a comparative analysis with other existing methods are studied to illustrate the simplicity and viability of the proposed fuzzy MCDM approach in the following section. Conclusion of this paper is given in the last part.

## Preliminaries

In this section, we first review the basic concepts of PFS, IVPFS, the SPA theory, fuzzy measure and Choquet integral.

### Interval-valued Pythagorean fuzzy set

#### Definition 1

[11]. Let *X* be a universe of discourse, then a Pythagorean fuzzy set (PFS) *P* in *X* can be denoted as

$$\begin{aligned} P = \{\left\langle x,t(x),m(x)\right\rangle | x\in X\}, \end{aligned}$$

where $t(x):X \rightarrow [0,1]$ , $m(x): X \rightarrow [0,1]$, $0\le (t(x))^2+(m(x))^2\le 1$ for all $x \in X.$ The *t*(*x*) is the membership degree and *m*(*x*) is the non-membership degree of *x*, respectively, and $\pi _p(x) = \root \of {1-(t(x))^2-(m(x))^2}$ is said to be the hesitancy degree of *x* to *P*. For convenience, $\left\langle t_p,m_p\right\rangle$ is called a Pythagorean fuzzy number (PFN) by Zhang and Xu [29] with $0\le t_p,m_p \le 1$ and $0\le t_p^2+m_p^2\le 1$.

#### Definition 2

[15]. Let *X* be a universe of discourse. An interval-valued Pythagorean fuzzy set (IVPFS) *P* in *X* is defined as

$$\begin{aligned} P = \{\left\langle x,[t_p^L(x),t_p^U(x)],[m_p^L(x),m_p^U(x)]\right\rangle |x\in X\}, \end{aligned}$$

where $$[t_p^L(x),t_p^U(x)] \subseteq [0,1]$$ and $$[m_p^L(x),m_p^U(x)] \subseteq [0,1]$$ such that $$0\le (t_p^U(x))^2+(m_p^U(x))^2\le 1$$ for all $$ x \in X$$. For convenience, an IVPFS can be expressed by an interval-valued Pythagorean fuzzy number (IVPFN) $$P=\left\langle [a,b],[c,d]\right\rangle $$ with the constrains $$[a,b] \subseteq [0,1],[c,d] \subseteq [0,1] $$, and $$b^2+d^2 \le 1,$$ and $$\pi _{p} = [\sqrt{1-b^2-d^2},\sqrt{1-a^2-c^2}]$$ is denoted the hesitation interval index of *P*. Specially, for any $$x \in X$$, if $$a=b=t_p,c=d= m_p $$, an IVPFN can reduce to a PFN $$P = \left\langle t_p, m_p \right\rangle $$.

#### Definition 3

[15]. Let $P=\left\langle [a,b],[c,d]\right\rangle$ be an IVPFN, a score function *T* of *P* can be shown as

1 $$\begin{aligned} T(P) = \frac{a^2+b^2-c^2-d^2}{2}, \qquad T(P)\in [-1, 1], \end{aligned}$$

and an accuracy function of IVPFN *P* can be shown as

2 $$\begin{aligned} V(P) = \frac{a^2 + b^2 + c^2 + d^2}{2}, \qquad V(P) \in [0,1]. \end{aligned}$$

Suppose there are two IVPFNs $P_1=\left\langle [a_1,b_1],[c_1,d_1]\right\rangle$ and $P_2=\left\langle [a_2,b_2],[c_2,d_2]\right\rangle$, then

- $T(P_1) > T(P_2) \Rightarrow P_1 \succ P_2$( ’$\succ$’ refer ’preferred to’);
- $$T(P_1) =T(P_2)$$, and
- $$V(P_1) > V(P_2) \Rightarrow P_1 \succ P_2$$ ;
- $V(P_1) = V(P_2) \Rightarrow P_1 \sim P_2$ ( ’$\sim$’ refer ’equivalent to’).

Especially, if $P_{max} = \left\langle [1, 1], [0, 0]\right\rangle$, then $T(P_{max}) = 1$ and $V(P_{max})=1$; if $P_{min} = \left\langle [0, 0], [1, 1]\right\rangle$, then $T(P_{min}) = -1$ and $V(P_{max})=1$.

#### Definition 4

[28]. An improved score function *R* of an IVPFN $P=\left\langle [a,b],[c,d]\right\rangle$ is denoted by

3 $$\begin{aligned} \begin{aligned} R(P) =&\frac{(a^2-c^2) \left( 1+\sqrt{1-b^2-d^2}\right) }{2}\\&+\frac{(b^2-d^2)\left( 1+\sqrt{1-a^2-c^2}\right) }{2}. \end{aligned} \end{aligned}$$

It is clear that $R(P)\in [-1, 1]$. If $P_1=\left\langle [a_1,b_1],[c_1,d_1]\right\rangle$ and $P_2=\left\langle [a_2,b_2],[c_2,d_2]\right\rangle$ are two IVPFNs, then an order between $P_1$ and $P_2$ can be denoted as follows:

$$\begin{aligned} \begin{aligned} (i)&~R(P_1) > R(P_2) \Rightarrow P_1 \succ P_2.\\ (ii)&~R(P_1) = R(P_2) \Rightarrow P_1 \sim P_2. \end{aligned} \end{aligned}$$

#### Definition 5

[12]. Let $P=\left\langle [a,b],[c,d]\right\rangle$ be an IVPFN. An improved accuracy function *C* of *P* is denoted by

4 $$\begin{aligned} C(P) = \frac{a^2\left( 1+\sqrt{1-b^2-d^2}\right) +b^2\left( 1+\sqrt{1-a^2-c^2}\right) }{2}\nonumber \\ \end{aligned}$$

where $C(P)\in [0, 1]$. Let $P_1=\left\langle [a_1,b_1],[c_1,d_1]\right\rangle$ and $P_2=\left\langle [a_2,b_2],[c_2,d_2]\right\rangle$ be two IVPFNs, then the following order holds:

$$\begin{aligned} \begin{aligned} \mathrm{(i)}&C(P_1) > C(P_2)\Rightarrow P_1 \succ P_2.\\ \mathrm{(ii)}&C(P_1) =C(P_2) \Rightarrow P_1 \sim P_2. \end{aligned} \end{aligned}$$

### Set pair analysis and connection numbers

#### Definition 6

[31]. Let $L(S_1,S_2)$ is a set pair consisting of two sets $S_1$ and $S_2$ for a given problem *G* . Suppose that there are total *N* features, *I* identical features , *C* contrary features , and $D=N-I-C$ discrepancy features in the problem *G*, respectively. The connection number (CN) $\mu$ of the set pair $L(S_1,S_2)$ for the problem *G* is defined as

5 $$\begin{aligned} \mu =a+bi+cj, \end{aligned}$$

where $a = \frac{I}{N}, b =\frac{D}{N}$ and $c=\frac{C}{N}$ show ’identity’, ’discrepancy’, and ’contrary’ degree between $S_1$ and $S_2$, respectively. It is clear that $0\le a \le 1$, $0 \le b \le 1$, $0 \le c \le 1,$ and $a+b+c=1$. In general, $i\in [-1,1]$ is the discrepancy coefficient; $j=-1$ is the contrary coefficient.

#### Definition 7

[37]. For any two CNs $\mu _1 = a_1 +b_1i +c_1j$ and $\mu _2 = a_2+b_2i+c_2j$ defined by Eq. (5), the following order between $\mu _1$ and $\mu _2$ holds:

$$\begin{aligned} \begin{aligned} \mathrm{(i)}&\quad \mu _1 =\mu _2 \Leftrightarrow a_1= a_2,b_1 =b_2,c_1 =c_2;\\ \mathrm{(ii)}&\quad a_1\ge a_2 \mathrm ~and ~ b_1 \le b_2 \Rightarrow \mu _1 \ge \mu _2. \end{aligned} \end{aligned}$$

#### Definition 8

[42]. For two CNs $\mu _1 = a_1 +b_1i +c_1j$ and $\mu _2 = a_2+b_2i+c_2j$ defined by Eq. (5), a score function $S_{F_1}$ of $\mu _k (k = 1,2)$ can be denoted by

6 $$\begin{aligned} S_{F_1}(\mu _k)= \left\{ \begin{array}{l} \begin{aligned} &{}(a_k-c_k)(1-b_k),&{} \qquad &{}if&{} a_k \ne c_k; \\ &{}a_k(1+b_k), &{} \qquad &{}if&{} a_k = c_k, \end{aligned} \end{array} \right. \end{aligned}$$

where $S_{F_1}(\mu _k)\in [-1,1]$. In addition, the following order between $\mu _1$ and $\mu _2$ holds:

$$\begin{aligned} \begin{aligned}&\mathrm{(i)}&S_{F_1}(\mu _1)> S_{F_1}(\mu _2)&\Rightarrow \mu _1 \succ \mu _2;\\&\mathrm{(ii)}&S_{F_1}(\mu _1)< S_{F_1}(\mu _2)&\Rightarrow \mu _1 \prec \mu _2;\\&\mathrm{(iii)}&S_{F_1}(\mu _1)= S_{F_1}(\mu _2)&\Rightarrow \mu _1 \sim \mu _2. \end{aligned} \end{aligned}$$

### Fuzzy measure and Choquet integral

In the MCDM process, the criteria usually have interactions with each other [43]. To illustrate these interactions, a fuzzy measure (or a non-additive measure) of the criteria is proposed by Sugeno [44].

#### Definition 9

[44]. Let *X* is a universe of discourse. A fuzzy measure $\mu$ on *X* is a set function $\mu : \pounds (X)\rightarrow [0,1]$, satisfying the following conditions:

$$\begin{aligned} \begin{aligned} \mathrm{(i)}~&\, \mu (\varnothing )=0, \mu (X)=1;\\ \mathrm{(ii)}~&\, A \subseteq B \Rightarrow \mu (A)\le \mu (B), \forall A,B\in \pounds (X),\\ \end{aligned} \end{aligned}$$

where $\pounds (X)$ is the power of universe *X*.

#### Definition 10

[45]. A $\lambda$-fuzzy measure $g_\lambda$ on a finite set $X=\{X_1, X_2, \ldots , X_n\}$ satisfies the following conditions:

7 $$\begin{aligned} \begin{aligned}&\mathrm{(i)} A_1, A_2 \in \pounds (X), A_1\cap A_2={\varnothing },A_1\cup A_2 \ne X\\&\quad \Rightarrow g_\lambda (A_1\cup A_2)=g_\lambda (A_1)+g_\lambda (A_2)+\lambda g_\lambda (A_1)g_\lambda (A_2) \end{aligned}\nonumber \\ \end{aligned}$$

8 $$\begin{aligned} \mathrm{(ii)} ~\frac{1}{\lambda }\left( \prod \limits _{i=1}^{n}[1+\lambda g_\lambda (X_{i})]-1\right) =1, \end{aligned}$$

where $$\lambda \in [-1, \infty )$$ but $$ \lambda \ne 0$$.

From Eq. (8), we can obtain

9 $$\begin{aligned} \prod \limits _{i=1}^{n}[1+\lambda g_\lambda (X_{i})]=\lambda +1. \end{aligned}$$

Therefore, $\lambda$ can be uniquely determined by all $g_\lambda (X_i) (i=1,2,\cdots ,n)$ and the Eq. (9).

#### Definition 11

[46] The discrete Choquet integral of function $f: X \rightarrow R^+$ with respect to a fuzzy measure $\mu$ on *X* can be defined by

10 $$\begin{aligned} C_{\mu }(f)=\sum \limits _{i=1}^{n}(\mu (A_{(i)})-\mu (A_{(i+1)}))\cdot f(X_{(i)}) \end{aligned}$$

where the subscript $({(1)}, {(2)}, \cdots , {(n)})$ indicates a permutation on *X* such that $f(X_{(1)})\le f(X_{(2)})\le \cdots \le f(X_{(n)})$ and $A_{(1)}=\{X_{(1)}, X_{(2)}, \cdots ,X_{(n)}\}$,$A_{(2)}=\{X_{(2)}, X_{(3)}, \cdots , X_{(n)}\},\cdots ,$ $A_{(i)}= \{X_{(i)},X_{(i+1)}\cdots , X_{(n)} \},\cdots , A_{(n)}=\{X_{(n)}\}, A_{(n+1)}= \varnothing$.

## Ranking method for IVPFNs based on SPA

In this section, we deal with the issue of ranking IVPFNs from a new perspective. First, based on the SPA theory, we propose a method to convert IVPFNs into CNs by taking the hesitation interval index and Pythagorean property into consideration simultaneously. Then an improved score function of CNs is given in the following part to make the ranking order of IVPFNs more in line with reality in fuzzy MCDM process.

### Conversion from IVPFNs into CNs

Through the literature, we have three findings as follows:

(i) The membership degree and non-membership degree of IVPFN are very close to the identity degree and contrary degree of CN respectively;
(ii) The hesitation interval index of IVPFN is an important influence factor for MCDM problems with interval-valued Pythagorean fuzzy information.
(iii) Since IVPFNs are the generalization of IVIFNs, the Pythagorean property should be fully considered to avoid the lack of uncertain information under interval-valued Pythagorean fuzzy environment as much as possible.

In view of the above three findings, we introduce the following definition of conversion from IVPFNs into CNs.

#### Definition 12

For an IVPFN $P = \left\langle [a,b],[c,d] \right\rangle$ with $0\le a\le b\le 1$,$0\le c\le d\le 1$ and $b^2+d^2\le 1$, the CN $\mu _p$ of *P* can be determined by

11 $$\begin{aligned} \mu _p = a_p+b_pi+c_pj, \end{aligned}$$

where

12 $$\begin{aligned} a_p= \frac{a^2\sqrt{2-b^2-d^2}+b^2\sqrt{2-a^2-c^2}}{2}, \end{aligned}$$

13 $$\begin{aligned} c_p= \frac{c^2\sqrt{2-b^2-d^2}+d^2\sqrt{2-a^2-c^2}}{2}, \end{aligned}$$

and

14 $$\begin{aligned} b_p = 1-a_p-c_p.~~~~~~~~~~~~~~~~~~~~~~~~~~~~~ \end{aligned}$$

#### Theorem 1

Let $P = \left\langle [a,b],[c,d] \right\rangle$ be an IVPFN with $0\le a\le b\le 1$, $0\le c\le d\le 1$ and $b^2+d^2\le 1$. Then the *CN* $\mu _p = a_p+b_pi+c_pj$ of *P* given by Eqs. (12–14) in Definition 12 is a rational CN.

*Proof* For an IVPFN $P = \left\langle [a,b],[c,d] \right\rangle$, to prove that the CN $\mu _p = a_p+b_pi+c_pj$ given by Eqs. (12–14) is a rational CN, we need to prove that $0\le a_p \le 1,0\le b_p \le 1$ and $0\le c_p \le 1$. Since $a_p \ge 0, c_p \ge 0$, then $a_p + c_p \ge 0$. In view of $b_p = 1-a_p-c_p$, we just need to prove $a_p+c_p \le 1$.

In fact, since $0\le a^2+c^2 \le b^2+d^2 \le 1$, then

$$\begin{aligned} a_p +c_p= & {} \frac{a^2\sqrt{2-b^2-d^2}+b^2\sqrt{2-a^2-c^2}}{2}\\&+ \frac{c^2\sqrt{2-b^2-d^2}+d^2\sqrt{2-a^2-c^2}}{2}\\= & {} \frac{(a^2+c^2)\sqrt{2-b^2-d^2}+(b^2+d^2)\sqrt{2-a^2-c^2}}{2}\\\le & {} \frac{(a^2+c^2+b^2+d^2)}{2}\sqrt{2-a^2-c^2} \\= & {} \left( \sqrt{\frac{(a^2+c^2+b^2+d^2)}{2}}\right) ^2\sqrt{2-a^2-c^2}\\\le & {} \left( \frac{\frac{(a^2+c^2+b^2+d^2)}{2}.2+2-a^2-c^2}{3}\right) ^\frac{3}{2}\\= & {} \left( \frac{2+b^2+d^2}{3}\right) ^\frac{3}{2} \le 1^\frac{3}{2} = 1 \end{aligned}$$

The above result is obtained using Mean inequality $\big ($i.e.$\frac{{x_1}^2+{x_2}^2+{x_3}^2}{3}\ge \root 3 \of {{x_1}^2{x_2}^2{x_3}^2}, \forall x_1,x_2,x_3 \ge 0$ $\big )$. Since $0\le a_p+c_p \le 1$ as proved above, then $0 \le a_p\le 1$ and $0 \le c_p \le 1$. Recall that $b_p = 1-a_p-c_p$ , we also obtain $0\le b_p \le 1$.$\square$

Obviously, for the largest IVPFN $P_{max} = \left\langle [1, 1], [0, 0]\right\rangle$, the CN of $P_{max}$ is $\mu _{p_{max}} = 1+0i+0j$; for the smallest IVPFN $P_{min} = \left\langle [0, 0], [1, 1]\right\rangle$, the CN of $P_{min}$ is $\mu _{p_{min} } = 0+0i+1j$; for $P = \left\langle [0,0],[0,0]\right\rangle$, the CN of *P* is $\mu _p = 0+i+0j$.

In the following, a simple example is given to show how to calculate the CN of an IVPFN.

#### Example 1

There are two IVPFNs: $P_1 = \left\langle [0.3, 0.6],[0.2,0.8]\right\rangle$ and $P_2 = \left\langle [\sqrt{0.20},\sqrt{0.35}],[\sqrt{0.3},\sqrt{0.6}]\right\rangle$. Then the CN of $P_1$ by Eqs. (12–14) are

$$\begin{aligned} \begin{aligned} a_{p_1}&= \frac{0.3^2\left( \sqrt{2-0.6^2-0.8^2}\right) +0.6^2\left( \sqrt{2-0.3^2-0.2^2}\right) }{2}\\&= 0.2911 \\ c_{p_1}&= \frac{0.2^2\left( \sqrt{2-0.6^2-0.8^2}\right) +0.8^2\left( \sqrt{2-0.3^2-0.2^2}\right) }{2}\\&= 0.4576 \\ b_{p_1}&= 1-a_{p_1}-c_{p_1} = 0.2513. \end{aligned} \end{aligned}$$

That is, $\mu _{p_1} = 0.2911 +0.2513 i+0.4576 j$. In the same way, we get the CN of $P_2$ is $\mu _{p_2} = 0.3168 +0.1621 i+0.5211 j$.

### Improved score function of CNs

Generally speaking, the methods for ranking CNs can be divided into two categories. One is to compare the identity degree and the discrepancy degree between CNs as mentioned before in Definition 7. However, by this method, only a partial order of IVPFNs can be obtained rather than a total order of IVPFNs. The other is to compare the values of score function of CNs to determine the ranking order of CNs as mentioned in Definition 8. However, as shown below, the score function in Definition 8 is unable to rank CNs correctly in some cases.

#### Example 2

Let $\mu _1 = 0.6+0i+0.4j, \mu _2 = 0.5 +0i +0.5j$ and $\mu _3 = 0.4 +0.2i +0.4j$ are three CNs. Using Eq. (6) in Definition 8, we get $S_{F_1}(\mu _1)=0.2, S_{F_1}(\mu _2)= 0.5$ and $S_{F_1}(\mu _3)=0.48$. Since $S_{F_1}(\mu _1)<S_{F_1}(\mu _3)<S_{F_1}(\mu _2)$, we obtain $\mu _1 \prec \mu _3 \prec \mu _2$. Obviously, this result is not only inconsistent with the result using Definition 7 ( by which we can get $\mu _3 \prec \mu _2 \prec \mu _1$ ), but also not in line with reality.

Therefore, to get a reasonable ordering of CNs, an improved score function of CNs is presented as follows.

#### Definition 13

Let $\mu = a+bi+cj$ be a CN denoted by Eq. (5). An improved score function $S_F$ of $\mu$ is defined as

15 $$\begin{aligned} S_F(\mu )= \left\{ \begin{array}{l} \begin{aligned} &{}a^2-c^2,&{} \qquad if~~a \ne c, \\ &{}a^2(1-a),&{} \qquad if~~a = c. \end{aligned} \end{array} \right. \end{aligned}$$

According to Definition 13, we can obtain the following properties of the proposed score function $S_F(\mu )$:

$$\begin{aligned} \begin{aligned} (P1 ) \mu&= 1 +0i +0j \Rightarrow S_F(\mu ) =1.\\ (P2 ) \mu&= 0 +1i +0j \Rightarrow S_F(\mu ) =0.\\ (P3 ) \mu&= 0 +0i +1j\Rightarrow S_F(\mu ) =-1.\\ (P4 ) \forall \mu&= a +bi +cj ( 0\le a, b, c\le 1,a+b+c=1)\\&\Rightarrow S_F(\mu ) \in [-1,1].\\ \end{aligned} \end{aligned}$$

Obviously, from above, we can obtain that the value of score function $S_F(\mu )$ of $\mu$ is between -1 and 1 for any CN $\mu = a +bi +cj$. In addition, the larger the value of $S_F(\mu )$, the more forward position of CNs will take in the ranking order. That is,

#### Definition 14

For two CNs $\mu _1 = a_1 +b_1i +c_1j$ and $\mu _2 = a_2+b_2i+c_2j$, the following order between $\mu _1$ and $\mu _2$ holds:

$$\begin{aligned} \begin{aligned}&\mathrm{(i)}&~S_F(\mu _1)> S_F(\mu _2)&\Rightarrow \mu _1 \succ \mu _2;\\&\mathrm{(ii)}&~S_F(\mu _1)< S_F(\mu _2)&\Rightarrow \mu _1 \prec \mu _2;\\&\mathrm{(iii)}&~S_F(\mu _1)= S_F(\mu _2)&\Rightarrow \mu _1 \sim \mu _2. \end{aligned} \end{aligned}$$

#### Example 3

If we apply the score function as given in Eq. (15) to the Example 2, we get $S_F(\mu _1)= 0.2$, $S_F(\mu _2)= 0.125$ and $S_F(\mu _3)=0.096$. Since $SF(\mu _1)>SF(\mu _2)>SF(\mu _3)$, we obtain that the ranking order is $\mu _1 \succ \mu _2 \succ \mu _3$. It is clear that the result is consistent with the result by Definition 7.

In the following, a few examples are presented to illustrate the advantage of proposed approach in this section.

#### Example 4

Suppose there are four different IVPFNs:

$$\begin{aligned} \begin{aligned} P_3 =&\left\langle [0.3,0.7],[0.3,0.7]\right\rangle ,\\ P_4 =&\left\langle [\sqrt{0.08},\sqrt{0.5}],[\sqrt{0.08},\sqrt{0.5}]\right\rangle ,\\ P_5 =&\left\langle [\sqrt{0.29},\sqrt{0.29}],[\sqrt{0.29},\sqrt{0.29}] \right\rangle ,\\ P_6 =&\left\langle [0.4,\sqrt{0.42}],[0.4,\sqrt{0.42}] \right\rangle .\\ \end{aligned} \end{aligned}$$

Acording to Defination 3, we get the value of score function for the four IVPFNs $$T(P_3) = T(P_4) = T(P_5) = T(P_6) = 0 $$ by Eq. (1), and the value of accuracy function for them $$V(P_3) = V(P_4) = V(P_5) = V(P_6) = 0.58$$ by Eq. (2). At the same time, according to Eq. (3) in Definition 4, we obtain $$R(P_3) = R(P_4) = R(P_5) = R(P_6) = 0 $$. Therefore, we can get $$P_3 \sim P_4\sim P_5 \sim P_6 $$. Obviously, $$P_3 \ne P_4\ne P_5 \ne P_6 $$.

In the following, we use the proposed approach in this paper to reorder the four IVPFNs. That is,

Step 1. Convert the four IVPFNs into CNs by Eqs. (12–14), then get
  $$\begin{aligned}&\qquad&\mu _{p_3} = 0.3760+0.2481i + 0.3760j;\\&\qquad&\mu _{p_4} = 0.3791 +0.2418i+0.3791j;\\&\qquad&\mu _{p_5} =0.3456+0.3089i+ 0.3456j;\\&\qquad&\mu _{p_6} = 0.3584+0.2833i+ 0.3584j. \end{aligned}$$
Step 2. Calculate the score value of CNs by Eq. (15), then get
  $$\begin{aligned}&\qquad&S_F(\mu _{p_3})= 0.0882,~ S_F(\mu _{p_4})=0.0892,\\&\qquad&S_F(\mu _{p_5})= 0.0782 , ~S_F(\mu _{p_6})=0.0824. \end{aligned}$$
Step 3. Compare the above score values, then get
  $$\begin{aligned} S_F(\mu _{p_5})< SF(\mu _{p_6})<S_F(\mu _{p_3}) < S_F(\mu _{p_4}). \end{aligned}$$

Therefore, the order of CNs is $\mu _5 \prec \mu _6\prec \mu _3 \prec \mu _4$. It is noticed that the order is also consistent with the result by Definition 7.

Hence, based on above steps, the ranking order of the four IVPFNs is $P_5 \prec P_6\prec P_3 \prec P_4$.

#### Example 5

Let $$P_7 = \left\langle [1,1],[0,0]\right\rangle $$ and $$P_8 =\left\langle [0.9,0.9],[0,0] \right\rangle $$ be two IVPFNs. According to the improved score function of IVPFNs defined in Eq. (4), we get $$C(P_7) = 1 $$ and $$C(P_8) = 1.1631 >1$$. Therefore, we obtain $$P_8 \succ P_7$$. However, it is obvious that $$P_8 \prec P_7$$, because $$P_7 =P_{max}$$ is the largest IVPFN as mentioned before. In the following, we use the proposed method in this section to reorder the two IVPFNs. Similar to the steps in Example 4,

Step 1. Convert $P_7$ and $P_8$ into CNs of them by Eqs. 12–14 and get
  $$\qquad \mu _{p_7} = 1+0i+ 0j,~~ \mu _{p_8} = 0.8836+0.1164i + 0j.$$
Step 2. According to Eq. (15), calculate the score value of CNs, then get
  $$ S_F(\mu _{p_7})=1,~~~~S_F(\mu _{p_8})=0.7807.$$
Step 3. Compare the above score values and get
  $$ S_F(\mu _{p_8}) < S_F(\mu _{p_7}).$$

Therefore, we obtain the order $P_7 \succ P_8$. It is clear that it is a reasonable order more in line with reality.

In fact, let ${\mathscr {P}} =\{ P_i =\left\langle [a_i,b_i],[c_i,d_i] \right\rangle | 0\le a_i\le b_i\le 1,0\le c_i\le d_i\le 1,b_i^2+d_i^2 \le 1, i\in \{1,2,\cdots ,n\}\}$ be the set of all IVPFNs. Then ${\mathbb {P}} =\{ P_i\in {\mathscr {P}} |a_i = c_i,b_i=d_i,i\in \{1,2,\cdots ,n\}\}$ is a special subset of ${\mathscr {P}}$. If $P_i,P_j \in {\mathbb {P}} (i \ne j )$ satisfy $a_i^2+b_i^2 = a_j^2+b_j^2$, by those previous methods [12, 28, 30], we get $P_i \sim P_j$ even if $P_i \ne P_j$ for any $i \ne j$. However, by the approach proposed in this section, a reasonable order between them can be obtained as shown in Example 4 above. In fact, the proposed approach in this section is suitable for all IVPFNs ranking problems in interval-valued Pythagorean fuzzy MCDM process. For example, by Eq. (15), the score values of CNs $\mu _{p_1}$ and $\mu _{p_2}$ in Example 1 are $S_F(\mu _{p_1})=-0.1247$ and $S_F(\mu _{p_2})=-0.1712$. Recall the score values of CNs obtained above in Example 4$--$5, we get $S_F(\mu _{p_2})<S_F(\mu _{p_1})<S_F(\mu _{p_5})< S_F(\mu _{p_6})<S_F(\mu _{p_3})< S_F(\mu _{p_4})<S_F(\mu _{p_8}) < S_F(\mu _{p_7})$, then we obtain the ranking order $P_2 \prec P_1 \prec P_5 \prec P_6 \prec P_3\prec P_4 \prec P_8\prec P_7$. That is, this proposed ranking approach is robust and it is more suitable for the ranking problem of IVPFNs than those previous methods. At the same time, as a ranking technique from a new perspective, the proposed approach is simple, effective and easy to implement.

## Interval-valued Pythagorean fuzzy multi-criteria decision making method

In this section, considering interactions among different criteria, we introduce a fuzzy MCDM method under interval-valued Pythagorean fuzzy environment by taking the advantages of proposed ranking technique above and Choquet integral.

Assume that, there are *m* alternatives, denoted by $A = \{A_1, A_2, . . . , A_m\}$, and *n* criteria, indicated as ${\mathbb {C}} = \{C_1, C_2, \cdots ,$ $C_n\}$. Let $P_{ij} = \left\langle [a_{ij},b_{ij}],[c_{ij},d_{ij}]\right\rangle \in {\mathscr {P}}$ be the estimation for alternative *i* on criteria *j*. Here $[a_{ij},b_{ij}]$ shows the degree of satisfaction, while $[c_{ij},d_{ij}]$ is the degree of dissatisfaction of alternative *i* with respect to criteria *j*, where $i=1,2,...,m,j=1,2,...,n$. In the following, to find the most desirable alternative in interval-valued Pythagorean fuzzy multi-criteria decision process, the specific steps of the proposed fuzzy MCDM method are given as follows:

Step 1. Construct and normalize the interval-valued Pythagorean fuzzy decision matrix $D(\widetilde{P}_{ij} )_{m\times n}$:
  $$\begin{aligned} \qquad D(\widetilde{P}_{ij} )_{m\times n}=\left( \begin{array}{c@{\quad }c@{\quad }c@{\quad }c} \widetilde{P}_{11}&{}\widetilde{P}_{12}&{}\dots &{}\widetilde{P}_{1n}\\ \widetilde{P}_{21}&{}\widetilde{P}_{22}&{}\dots &{}\widetilde{P}_{2n}\\ \vdots &{}\vdots &{}\ddots &{}\vdots \\ \widetilde{P}_{m1}&{}P_{m2}&{}\dots &{}\widetilde{P}_{mn} \end{array}\right) . \end{aligned}$$
  where
  16 $$\begin{aligned} \qquad \widetilde{P}_{ij}= \left\{ \begin{array}{l} \begin{aligned} &{}\left\langle [a_{ij},b_{ij}],[c_{ij},d_{ij}]\right\rangle ,&{}\qquad &{}if~&{} C_j \in {\mathbb {B}},\\ &{}\left\langle [c_{ij},d_{ij}],[a_{ij},b_{ij}] \right\rangle ,&{}\qquad &{}if &{}C_j \in {\mathbb {T}}, \end{aligned} \end{array} \right. \nonumber \\ \end{aligned}$$
  of which, ${\mathbb {B}}$ represents the benefit type criteria subset, and ${\mathbb {T}}$ is the cost type criteria subset.
Step 2. Convert the above matrix into a CNs matrix:
  $$\begin{aligned}\qquad&U( \mu _{ij})_{m\times n}\\ \qquad&\quad =\left( \begin{array}{c@{\quad }c@{\quad }c@{\quad }c} \mu _{11}&{}\mu _{12}&{}\dots &{}\mu _{1n}\\ \mu _{21}&{}\mu _{22}&{}\dots &{}\mu _{2n}\\ \vdots &{}\vdots &{}\ddots &{}\vdots \\ \mu _{m1}&{}\mu _{m2}&{}\dots &{}\mu _{mn} \end{array}\right) \\ \qquad&\quad =\left( \begin{array}{c@{\quad }c@{\quad }c@{\quad }c} a_{11}+b_{11}i+c_{11}j&{}\dots &{}a_{1n}+b_{1n}i+c_{1n}j\\ a_{21}+b_{21}i+c_{21}j&{}\dots &{}a_{2n}+b_{2n}i+c_{2n}j\\ \vdots &{}\ddots &{}\vdots \\ a_{m1}+b_{m1}i+c_{m1}j&{}\dots &{}a_{mn}+b_{mn}i+c_{mn}j \end{array}\right) , \end{aligned}$$
  where $\mu _{ij} = a_{ij}+b_{ij}i+c_{ij}j$ is the CN of $\widetilde{P}_{ij}$. In addition, $a_{ij},b_{ij}, c_{ij}$ are calculated by Eqs. (12–14) in Definition 12, respectively, for $i=1,2,\cdots ,m,j=1,2,\cdots ,n$.
Step 3. Calculate the value of score function $S_F(\mu _{ij})$ of $\mu _{ij}$ $(i=1,2,\cdots ,m;j=1,2,\cdots ,n)$ using Eq. (15) to obtain the score function matrix as follows:
  $$\begin{aligned}\qquad&S_F(\mu _{ij})_{m\times n}\\ \qquad&\quad =\left( \begin{array}{cccc} S_F(\mu _{11})&{}S_F(\mu _{12})&{}\dots &{}S_F(\mu _{1n})\\ S_F(\mu _{21})&{}S_F(\mu _{22})&{}\dots &{}S_F(\mu _{2n})\\ \vdots &{}\vdots &{}\ddots &{}\vdots \\ S_F(\mu _{m1})&{}S_F(\mu _{m2})&{}\dots &{}S_F(\mu _{mn}) \end{array}\right) .\end{aligned}$$
Step 4. Aggregate the above score values on each criterion to each alternative by Choquet integral. The specific implementation process is as follows:
Calculate the $\lambda$-fuzzy measure of each criterion set. In general, to obtain the $\lambda$-fuzzy measure of each criterion set, we should first get the weight, or the importance, of each criterion [43]. At present, there are three typical methods to determine the weight of criteria, i.e., the subjective method, the objective method and hybrid method [47]. No matter which method is used, the weight is obtained by a numerical value from 0 to 1 [46]. In this paper,we denote the weight of criteria *j* by $g_\lambda (C_j), j=1,\cdots ,n$. According to the Eq. (9), we get
  17 $$\begin{aligned} \quad \qquad \lambda +1=\prod \limits _{i=1}^{n}[1+\lambda g_\lambda {(C_j)}]. \end{aligned}$$
  Then we can obtain the parameter $\lambda$ by resolving Eq. (17) if we have get all $g_\lambda (C_j)(j=1,\cdots ,n)$ in some way. Based on the parameter $\lambda$, the fuzzy measure of each criteria set can be derived by Eq. (7).
Calculate the Choquet integral of the score values of CNs for each alternative. According to the above fuzzy measures, we can aggregate the score values of CNs for each alternative with respect to each criterion as follows:
  18 $$\begin{aligned} \qquad \quad C_{g_\lambda }(S_{F})_i= & {} \sum \limits _{j=1}^{n}(g_\lambda (A_{(j)})-g_\lambda (A_{(j+1)}))\nonumber \\&\cdot S_F(\mu _{i(j)} ) \end{aligned}$$
  for any $i \in \{1,2,\cdots , m \}$. Here the subscript $((1), (2), \cdots ,(n))$ is a permutation on *X* such that $S_F(\mu _{i(1)})\le S_F(\mu _{i(2)})\le \cdots \le S_F(\mu _{i(n)})$ and $A_{(1)}= \{C_{(1)},C_{(2)},\cdots ,C_{(n)}\},A_{(2)}=\{C_{(2)},C_{(3)}$ $\cdots , C_{(n)}\},\cdots , A_{(n)}=\{ C_{(n)}\}, A_{(n+1)}= \varnothing$.
Step 5. According to the aggregated values above, obtain a reasonable ranking order of all alternatives to find the most desirable one.

**Fig. 1:**
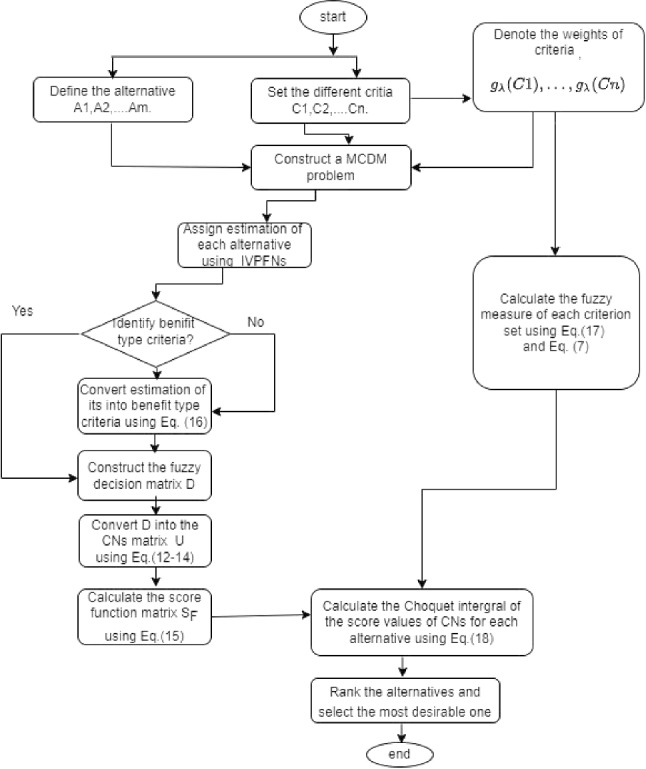
Flow chart of the proposed method

The complete flow chart of the proposed method is given in Fig. 1.

## Discussion and comparative study

To illustrate the convenience and feasibility of our proposed approach, an example about the online learning satisfaction survey is given at first. Then the relevant discussion and comparative analysis between the proposed approach and other existing approaches [12, 15, 28, 30, 43, 49] are provided to validate the performance of the proposed approach in the paper.

### Illustrative example

During the COVID-19 pandemic, a large number of Chinese universities implement large-scale online learning, which is a new model that can break the constraints of time and space for students. In the post-epidemic era, how to improve the effect of online learning model has become a very important topic. The online learning satisfaction survey is an important way that can help educators understand the real needs of students. The data in this section come from an online learning satisfaction survey conducted during the COVID-19 pandemic on a questionnaire survey platform [48]. By survey, it is found that the involved students can be divided into four categories according to the nature of their schools: research-oriented university ($A_1$), application-oriented university ($A_2$), higher vocational college ($A_3$), and other types ($A_4$). In this paper, we study the ranking order of online learning satisfaction for the four types of schools based on SPA and Choquet integral. We select four criteria for research, that is, the service of learning resource ($C_1$), the communication between teachers and students ($C_2$), the design of the course ($C_3$) and the communication between students ($C_4$). By analysis, it is found that the four criteria are not independent(or say there are interactions among them). For example, the more extensive the service of learning resource, and the more interesting the teacher’s design of course, the more active the communication between teachers and students, and vice versa. In addition, the communication between teachers and students can also improve the communication between students, and vice versa. Therefore, the decision-making steps are described as below:

(Step 1:) Through analysis, it is found that all four criteria are benefit-type. Each type of school $A_i (i = 1, 2, 3, 4)$ is given four evaluation values by their students which represent the degree of satisfaction on each criterion $C_j (j = 1, 2, 3, 4)$. The values are provided in the form of IVPFNs. Thus, the normalized Pythagorean fuzzy decision matrix $D(\widetilde{P}_{ij})_{4\times 4}$ can be obtained by Eq. (16):
  $$\begin{aligned} \qquad \quad D(\widetilde{P}_{ij})_{4\times 4} =\left( \begin{array}{cccc} C_1&{}C_2\\ \langle [0.5103, 0.7001],[0.3565, 0.4103]\rangle &{}\langle [0.4054,0.6043],[0.5002,0.6154]\rangle \\ \langle [0.6220, 0.8020],[0.2310, 0.4112]\rangle &{}\langle [0.6146,0.8000],[0.2020,0.3011]\rangle \\ \langle [0.8130,0.9200],[0.1012,0.2426]\rangle &{}\langle [0.5098,0.7046],[0.3134,0.5356]\rangle \\ \langle [0.4033, 0.6013],[0.3433, 0.5214]\rangle &{}\langle [0.5022,0.6780],[0.4033,0.6062]\rangle \\ &{}C_3&{}C_4\\ &{}\langle [0.6033,0.7523],[0.3109,0.4323]\rangle &{}\langle [0.7521, 0.9121],[0.2102,0.3501]\rangle \\ &{}\langle [0.7327,0.8567],[0.3006,0.3500]\rangle &{}\langle [0.7435, 0.8500],[0.3342, 0.5520]\rangle \\ &{}\langle [0.7452,0.9304],[0.2091,0.3012]\rangle &{}\langle [0.5090, 0.6000],[ 0.4042,0.8321]\rangle \\ &{}\langle [0.5021,0.7563],[0.2500,0.4432]\rangle &{}\langle [0.6431, 0.8074],[0.2405,0.4064]\rangle \\ \end{array}\right) \end{aligned}$$
  where the interval [0.5103, 0.7001] means the certain degree that alternative $A_1$(research-oriented universities) satisfies $C_1$ (the service of learning resource), and [0.3565, 0.4103] is the uncertain degree that the alternative $A_1$ dissatisfies $C_1$. The other values in $D(\widetilde{P}_{ij})_{4\times 4}$ have similar meanings.
(Step 2:) Convert the above matrix into the CNs matrix $U(\mu _{ij})_{4\times 4}$ by Eqs. (12–14), that is
  $$\begin{aligned} \,\quad \qquad&U(\mu _{ij})_{4\times 4} \\ \,\quad \qquad&\quad = \left( \begin{array}{ccc} C_1&{}C_2\\ 0.4620 +0.3575 i+0.1805 j&{}0.3220 +0.2994 i+0.3786 j\\ 0.6125 +0.2529 i+0.1347 j&{}0.6152 +0.3048 i+0.0800 j\\ 0.8336 +0.1271 i+0.0393 j&{}0.4614 +0.3007 i+0.2380 j \\ 0.3321 +0.4207 i+0.2471 j&{}0.4259 +0.2546 i+0.3194 j\\ C_3 &{}C_4\\ 0.5543 +0.2758 i+0.1699 j &{}0.7797 +0.1255 i+0.0948 j\\ 0.7170 +0.1629 i+0.1201 j &{}0.6901 +0.0788 i+0.2311 j\\ 0.7960 +0.1280 i+0.0760 j &{} 0.3522 +0.1335 i+0.5143 j\\ 0.5112 +0.3266 i+0.1622 j &{}0.6279 +0.2385 i+0.1336 j\\ \end{array}\right) \end{aligned}$$
(Step 3:) Convert the above matrix into the score function matrix $S_F(\mu _{ij})_{4\times 4}$ by Eq. (15):
  $$\begin{aligned}\qquad&S_F(\mu _{ij})_{4\times 4}\\ \qquad&\quad =\left( \begin{array}{cccc} C_1&{}C_2&{}C_3&{}C_4\\ 0.1809&{}-0.0396&{}0.2784&{}0.5989\\ 0.3570&{} 0.3724&{}0.4997&{}0.4228\\ 0.6934&{}0.1563&{} 0.6278&{}-0.1405\\ 0.04924&{}0.0794&{}0.2350&{}0.3765\\ \end{array}\right) \end{aligned}$$
(Step 4:) The fuzzy measures of all the four criteria can be calculated by the method in [47]. This is an objective method by training a lot of data from the questionnaire survey platform [48]. The fuzzy measure of each criterion is obtained as follows [47]:
  19 $$\begin{aligned} \qquad \qquad g_\lambda (\cdot )= & {} \{g_\lambda {(C_1)},g_\lambda {(C_2)},g_\lambda {(C_3)},g_\lambda {(C_4)}\}\nonumber \\= & {} \{0.35,0.25,0.2,0.18\} \end{aligned}$$
  Then, by Eq. (17), we get the parameter $\lambda =-0.64$. And by Eq. (7), the fuzzy measure of each criteria subset can be obtained in Table 1.
(Step 5:) Calculate the overall satisfaction degree of all types schools based on Choquet integral by Eq. (18) as follows:
  $$\begin{aligned} \qquad \qquad \qquad C_{g_\lambda }(S_{F}(A_1))= & {} 0.236854905172487 ,\\ \qquad \qquad \qquad C_{g_\lambda }(S_{F}(A_2))= & {} 0.407867581010157,\\ \qquad \qquad \qquad C_{g_\lambda }(S_F(A_3))= & {} 0.369070525859337,\\ \qquad \qquad \qquad C_{g_\lambda }(S_F(A_4))= & {} 0.171711061553457. \end{aligned}$$

Since $C_{g_\lambda }(S_{F}(A_4))\le C_{g_\lambda }(S_{F}(A_1))\le C_{g_\lambda }(S_{F}(A_3))$ $\le C_{g_\lambda }(S_{F}(A_2))$, the ranking order of alternatives is $A_4 \prec A_1 \prec A_3 \prec A_2$. That is, the students from $A_2$ (application-oriented university) have the highest degree of satisfaction towards online learning. This result is consistent with the result of questionnaire survey.

**Table 1:** The fuzzy measures of criteria

C	$g_\lambda {(C)}$	C	$g_\lambda {(C)}$
$\{C_1\}$	0.35	$\{C_2\}$	0.25
$\{C_3\}$	0.20	$\{C_4\}$	0.18
$\{C_1,C_2 \}$	0.63	$\{C_1,C_3\}$	0.56
$\{C_1,C_4\}$	0.50	$\{C_2,C_3\}$	0.58
$\{C_2,C_4\}$	0.43	$\{C_3,C_4\}$	0.48
$\{C_1,C_2,C_3\}$	0.75	$\{C_1,C_2,C_4\}$	0.70
$\{C_1,C_3,C_4\}$	0.78	$\{C_2,C_3,C_4\}$	0.74
$\{C_1,C_2,C_3,C_4\}$	1.00	$\varnothing$	0

**Table 2:** Comparative analysis for examples in “Illustrative example”

Methodology	Core idea of method	Ordering
Garg [12]	Improved accuracy function	$A_2 \succ A_3 \succ A_1 \succ A_4$
Zhang [15]	Closeness index	$A_2 \succ A_3 \succ A_1 \succ A_4$
Garg [28]	Improved score function	$A_2 \succ A_3 \succ A_1 \succ A_4$
Garg [30]	Novel accuracy function	$A_2 \succ A_3 \succ A_1 \succ A_4$
Khan [43]	TOPSIS	$A_2 \succ A_3 \succ A_1 \succ A_4$
Khan [49]	IVPFCIA	$A_3 \succ A_2 \succ A_1 \succ A_4$
Proposed	SPA	$A_2 \succ A_3 \succ A_1 \succ A_4$

### Discussion and comparison analysis

To illustrate the advantages of proposed approach in this paper, we conduct a brief discussion and a comparative analysis with some of the existing approaches [12, 15, 28, 30, 43, 49] under interval-valued Pythagorean fuzzy environment. These approaches corresponding to them are performed on the considered data and the rankings of alternatives which are summarized in Table 2. As shown in Table 2, it is easily observed that:

(i) The approach proposed in this paper, from a new perspective, that is, SPA, deals with fuzzy information in the interval-valued Pythagorean fuzzy multi-criteria decision-making problems. To our knowledge, Zhang [15] proposed a closeness index for IVPFNs and presented the closeness index-based ranking method (QUALIFLEX MCDM approach) for ranking IVPFNs. In addition, Garg [12, 28, 30] proposed a novel accuracy function or an improved score function for IVPFNs for solving MCDM problems under interval-valued Pythagorean fuzzy environment. Khan et al. [43, 49] introduced an extension of TOPSIS method and a fuzzy GRA method under interval-valued Pythagorean fuzzy environment. Essentially, they are still ranking IVPFNs based on score function and accuracy function of IVPFNs [15] before integration process by the Choquet integral. However, the main idea of the approach proposed in this paper is to convert IVPFNs into CNs using SPA theory before integrating the values of alternatives by Choquet integral. Comparing with the existing approaches, this idea of transformation is first given under the general interval-valued Pythagorean fuzzy environment in the literature. According to the decision-making processes and results in Table 2, the proposed approach in this paper not only can effectively solve the MCDM problems under interval-valued Pythagorean fuzzy environment, but also it is simple, convenient and easy to implement. Meanwhile, the result of our proposed approach is consistent with most existing approaches.
(ii) The approach proposed in this paper can deal with the interval-valued Pythagorean fuzzy multi-criteria decision-making problems not only under the general interval-valued Pythagorean fuzzy environment, but also in some special cases which cannot be solved by existing methods [15, 28, 43, 49]. For example, suppose it is still the online learning satisfaction survey question in the above section and the fuzzy measure of all four criteria is also given by Eq. 19. Suppose that the evaluation values of an alternative $$A_0$$ on four criteria are $$P_3,P_4,P_5,P_6$$ in Example 4, respectively. Since the values of score function for the four IVPFNs are $$T(P_3) = T(P_4) =T(P_5) = T(P_6) = 0 $$ and the accuracy functions of them are $$V(P_3) = V(P_4) = V(P_5) = V(P_6) = 0.58$$, and the closeness index $${\mathscr {P}}(P_3) = {\mathscr {P}}(P_4) = {\mathscr {P}}(P_5) = {\mathscr {P}}(P_6) = 0.5$$ (for details,see [15]), we can get $$P_3 \sim P_4\sim P_5 \sim P_6 $$. In this case, the Choquet integral cannot be performed, because IVPFNs cannot be ranked reasonably. Therefore, the approaches [15, 28, 43, 49] are invalid. However, according to the proposed approach in this paper, we get $$S_F(\mu _{p_3})= 0.0882, S_F(\mu _{p_4})=0.0892, S_F(\mu _{p_5})= 0.0782 , $$ $$S_F(\mu _{p_6})=0.0824$$, then $$P_5 \prec P_6\prec P_3 \prec P_4 $$ as mentioned in Example 4. By simple calculation, we get the Choquet integral of the alternative $$A_0$$ is $$C_{g_\lambda }(A_0) = 0.0782*(g_\lambda (C_1,C_2,C_3,C_4)-g_\lambda (C_1,C_2,C_4))+0.0824*(g_\lambda (C_1,C_2,C_4)-g_\lambda (C_1,C_2)) +0.0882 *(g_\lambda (C_1,C_2)-g_\lambda (C_2))+ 0.0892*g_\lambda (C_2) =0.0782 *(1-0.7)+0.0824*(0.7-0.63)+0.0882*(0.63-0.25)+ 0.0892*0.25= 0.0850. $$ Therefore, the proposed approach in this paper is robust and can obtain the overall satisfaction evaluation from a new perspective.
(iii) The approach proposed in this paper takes the interactions among criteria into account using Choquet integral in MCDM under interval-valued Pythagorean fuzzy environment. While the existing approaches [12, 28, 30] can only deal with the MCDM problem in which the relationships among criteria are assumed to be independent in advance. At the same time, from the result of comparison which is shown in Table 2, we find that the result of our proposed approach is consistent with the method made by Zhang [15] and Khan [43] which takes into account the interaction between the criteria in MCDM under interval-valued Pythagorean fuzzy environment.

## Conclusions

IVPFS is a useful tool for dealing with uncertain information in MCDM process, while the SPA theory has the advantage of combining the certainty and uncertainty into a unified way. In view of these advantages, we propose a method to convert IVPFNs into CNs and introduce an improved score function of CNs for the first time. Furthermore, taking into account the uncertainty in decision-making and interactions between criteria simultaneously, we propose a fuzzy MCDM approach based on SPA and Choquet integral to get the overall degree of satisfaction for each alternative. The key contribution of this study is

1. a novel technique of converting IVPFNs into CNs is proposed considering the uncertainty and Pythagorean property of IVPFNs;
2. an improved score function of CNs is developed to overcome the shortcomings of previous formulas;
3. an effective ranking method for IVPFNs is introduced from a new perspective for the first time;
4. an interval-valued pythagorean fuzzy multi-critria decision-making method based on SPA and Choquet intergral is proposed by considering the interactions among criteria.

From examples shown above, we can see that the proposed approach successfully overcomes the drawbacks presented in [12, 15, 28, 30, 42, 43, 49]. The proposed approach provides us with a very useful way to deal with MCDM problems in the IVPFSs context. At the same time, examples show that the proposed approach is simple and easy to implement in interval-valued Pythagorean fuzzy MCDM process.

However, the tendency of decision-makers towards fuzzy indexes (the membership, non-membership or hesitation) of IVPFSs has a significant impact on the outcome of the decision-making in many fields. Here the tendency refers to the decision-makers’ attitudes, habits, knowledge or experience, etc. In this paper, we failed to take into account the tendency of decision makers towards different fuzzy indexes of IVPFSs in the process of converting IVPFNs into CNs.In the future, on one hand, we will consider the tendency of decision makers in the process of converting IVPFNs into CNs, and on the other hand, we will also apply the proposed approach to more fields, such as pattern recognition, the security of industrial control, the big data of education, etc.
